# Biocontrol of Invasive Conical Snails by the Parasitoid Fly *Sarcophaga villeneuveana* in South Australia 20 Years after Release

**DOI:** 10.3390/insects12100865

**Published:** 2021-09-24

**Authors:** Kate A. Muirhead, Kym D. Perry

**Affiliations:** 1 South Australian Research and Development Institute, Adelaide, SA 5001, Australia; 2 School of Agriculture, Food and Wine, The University of Adelaide, Adelaide, SA 5005, Australia

**Keywords:** *Cochlicella acuta*, *Cochlicella (Prietocella) barbara*, *Sarcophaga penicillata*

## Abstract

**Simple Summary:**

Two invasive conical snail species are major pests of pastures and grain crops in Australia. In 2000, a parasitoid fly, *Sarcophaga villeneuveana*, was sourced from the snails’ native Mediterranean range and introduced on the Yorke Peninsula, South Australia, for biological control of the conical snail *Cochlicella acuta*. The fly successfully established in the region but assessments of the fly’s impact in different snail habitats were limited. Twenty years on, four field surveys were performed over two years to measure its geographic spread and parasitism rates on *C. acuta* and the small pointed snail, *C. barbara*. In total, >88,000 snails were collected using standardised sampling methods to investigate the parasitism of host snails in different habitats. The fly was found at 13 of 19 sampled sites up to 34 km from nursery release sites. Total parasitism rates of suitably sized snails (≥5 mm shell height) were ≈3% for both *C. acuta* and *C. barbara*. Rates were higher in *C. acuta* (5.4%) and *C. barbara* (15.2%) in exposed habitats above ground level. Parasitism rates up to 48% in *C. acuta* and 29% in *C. barbara* at sites near flowering vegetation suggested that the fly benefits from floral resources.

**Abstract:**

Two conical snail species introduced to Australia from the Mediterranean region during the 20th century are major pests of pastures and grain crops. In 2000, a parasitoid fly, *Sarcophaga villeneuveana*, was introduced into South Australia for biocontrol of the conical snail, *Cochlicella acuta*. The fly successfully established in the region but assessments of its impact in different snail aestivation microhabitats were limited. Twenty years on, field surveys were conducted to assess the geographic distribution and parasitism rates of *S. villeneuveana* on conical snails in the Yorke Peninsula region. Nineteen sites were sampled on four occasions in January and April of both 2019 and 2020. In total, >85,600 *C. acuta* and >2400 *C. barbara* were collected from cryptic (ground or plant refuge) and exposed (open ground or elevated substrate) aestivation habitats and assessed for parasitism. The fly was detected at 13 of 19 sampled sites up to 34 km from nursery release sites. Total parasitism rates of suitably sized snails (≥5 mm shell height) were 2.9% for *C. acuta* and 3.4% for *C. barbara*. Maximum parasitism rates of 48% for *C. acuta* and 29% for *C. barbara* were found at sites adjacent to spring- and summer-flowering native vegetation. Across 13 sites, parasitism rates were higher for *C. acuta* (5.4%) and *C. barbara* (15.2%) in exposed habitats above ground level. However, only 34% of *C. acuta* and 14% of *C. barbara* were found in elevated habitats as most snails were found in cryptic refuges. There was a seasonal decline in abundance of *C. acuta* (66%) and *C. barbara* (45%) between January and April, suggesting natural mortality. Although the overall impact of the fly is limited, high parasitism rates in local environments with flowering resources indicates the potential to enhance biocontrol of both invasive conical snail species.

## 1. Introduction

Four Mediterranean snail species introduced into southern Australia are major pests of pastures and grain crops: the conical snails, *Cochlicella acuta* (Müller) and *Cochlicella (Prietocella) barbara* (Linnaeus) (Geomitridae), and the round snails, *Theba pisana* (Müller) (Helicidae) and *Cernuella virgata* (da Costa) (Geomitridae) [1,2]. They feed on seedlings in autumn and winter, foul pastures, and contaminate grain harvests by aestivating on the heads, pods and stalks of cereals and legumes [1,2]. All four species have expanded their Australian distributions via human transport and become locally abundant in agricultural areas. The conical snail, *C. acuta*, was first discovered on Yorke Peninsula (YP) in South Australia in 1953 and the small pointed snail, *C. barbara*, was first detected in 1921 [1]. Both species have spread across southern and southwestern Australia [3,4].

Integrated management of snails in Australian crops and pastures involves a costly program of molluscicidal baiting, managing crop stubble (e.g., rolling, grazing, cabling, burning) and weed refuges, minimising harvest contamination by snails and postharvest grain cleaning [5]. Conical snails are less susceptible than round snails to most controls due to their more cryptic habits, including a tendency to aestivate in refuges under rocks, logs and inside plants [6], and difficulty in post-harvest separation of snails from physically similar grains [5].

During the 1990s, the Australian Commonwealth Scientific and Industrial Research Organisation (CSIRO) surveyed the native range of Mediterranean pest snails for natural enemies to implement in classical biological control programs [7,8,9,10,11,12]. Among several species from the dipteran families Sciomyzidae and Sarcophagidae considered as potential biocontrol agents, only *Sarcophaga villeneuveana* (then known as *S. penicillata*) (Diptera: Sarcophagidae) displayed sufficient host specificity on pest snails, in particular *C. acuta*, and was cleared for release in Australia [12,13].

Adult *S. villeneuveana* are active from approximately early spring to mid-autumn. They attack aestivating conical snails ≥5 mm in shell height [7,14]. Adults mate within hours after emergence and females are ovoviviparous, depositing a live larva near the shell aperture. The larva crawls inside the shell and feeds on the snail flesh, eventually killing it. After ≈7 days, the larva pupates inside the shell and emerges ≈18 days later as an adult fly [7]. Approximately 5–6 generations are possible during spring and summer [7]. Flies enter diapause in late autumn and overwinter in the pupal stage inside the snail shell.

**Figure 1:**
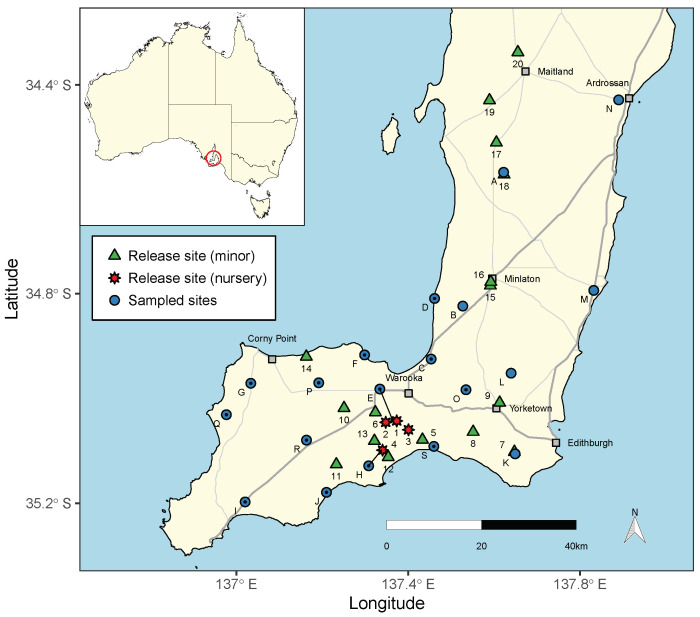
Geographic map of the Yorke Peninsula, South Australia, showing sites where *S. villeneuveana* was released during 2000–2004 and locations sampled in 2019–2020. Multiple fly releases occurred at nursery sites (red stars) and single releases occurred at minor sites (green triangles). Sites where the fly was present (blue circles with a black dot) or not found (blue circles) are shown.

A classical biological control program targeting *C. acuta* was initiated in 2000 in South Australia [13] using *S. villeneuveana*. The fly was collected from the Montpellier region in southern France, imported and mass-reared at the South Australian Research and Development Institute (SARDI). Between 2000 and 2004, >10,000 *S. villeneuveana* adults were released at 20 sites on the YP and two sites on the Limestone Coast [12,13] (Figure 1). Sequential releases were conducted at four "nursery" sites on the southern YP in the summers of 2002–03 and 2003–04 (n≈ 1000–2000 flies per site) [13]. Single releases were made at additional locations in the summers of 2000–01, 2001–02 and 2003–04 (n≈ 200–400 flies per site) (Figure 1).

Subsequent surveys on the Yorke peninsula in 2005, 2007–08 and 2016–17, found that parasitism rates of *C. acuta* were generally <2%, with a maximum of 17–20% reported (SARDI, unpub.). These surveys mainly assessed snails ≥7 mm on substrates elevated off the ground (e.g., stubble, fenceposts) and did not include other microhabitats or snails 5–7 mm that are also susceptible to attack [14]. The tendency of conical snails to aestivate in cryptic as well as exposed habitats and higher parasitism rates of elevated snails [7] suggested that overall parasitism rates could have been overestimated in past surveys. *Sarcophaga villeneuveana* has since been reared from *C. barbara* collected in Morroco [15] and South Australia (SARDI unpub.), showing that this species is also a suitable host.

The aims of this study were to investigate the geographic distribution of *S. villeneuveana* on the YP and the parasitism rates of suitable *C. acuta* and *C. barbara* (≥5 mm) in different microhabitats. Parasitism rates were compared between coexisting populations of *C. acuta* and *C. barbara* to assess the relative host suitability of these species for *S. villeneuveana*. Surveys were conducted on the YP in 2019 and 2020 in mid-summer (January) and at the end of the active period of *S. villeneuveana* in autumn (April) to assess temporal changes in parasitism rates. Since conical snails do not breed over summer [6], parasitism rates should increase with successive fly generations throughout their active season. Parasitism was examined in exposed and cryptic snail aestivation microhabitats to determine ecological specialisation of *S. villeneuveana*. This work assessed the impact from the initial fly releases after 20 years and provides baseline data for future attempts to improve biocontrol of conical snails.

## 2. Methods

### 2.1. Field Sampling and Data Collection

Field sampling in agricultural areas on the Yorke Peninsula, South Australia, was conducted on four occasions: 21–24 January and 9–12 April 2019, and 20–23 January and 21–23 April 2020. Fourteen sites were sampled in January 2019 and an additional five (19 in total) were sampled on all subsequent occasions (Figure 1). In April 2020, site K was within an area recently burned by fire and no live snails were found.

Standardised sampling was performed using a belt transect method and timed searches. At each site, four 25 × 2 m transects were sampled along roadside verges bordering paddocks with cereals, canola, legumes, or pasture. In each transect, *C. acuta* and *C. barbara* of suitable size for parasitism (shell height ≥ 5 mm from apex to aperture [7,14]) were collected from four microhabitats: (1) substrates elevated above ground level, such as on plants, stubble and fence posts (elevated); (2) under the base of plants and inside grass tussocks (plant refuge); (3) under refuges at ground level, such as logs and rocks (ground refuge); and (4) within quadrats at ground-level (quadrat). Elevated, plant refuge and ground refuge habitats were sampled by searching each transect and nearby areas for a period of five minutes per habitat. A 30 × 30 cm quadrat was randomly placed at five-metre intervals in each transect (five quadrats per transect) and all suitable snails were collected. Snails were returned to SARDI laboratories in Adelaide and maintained in plastic containers with mesh lids at 20–24 ${}^{\circ }C$, 14 light/10 h dark, and 30–40% relative humidity for at least two weeks to allow flies to emerge. After rearing, snails clearly dead for longer than the rearing period were excluded and the remaining snails were visually examined for evidence of parasitism. Assessments based solely on fly emergence underestimate attack rates and mortality [14]; therefore, evidence of parasitism included the presence of a fly larva or pupa, an open fly pupal case or a fly inside the shell (Table 1 and Table 2). Snails not clearly alive or in the above categories were dissected and examined. Parasitism rates were calculated by dividing the number of snails with evidence of parasitism by the total number of suitable snails collected. Snails that died of unknown causes after collection were included in the total.

### 2.2. Data Analysis

Count data from four transects were pooled prior to analysis, providing a single sample from each habitat per site and sampling occasion. Statistical analysis was performed using R version 4.0.5 [16] and plots visualised using ggplot2 [17]. Geographic distances between field sites were calculated using R package geosphere version 1.5–10 [18].

Snail abundance and parasitism data were analysed using generalised linear mixed models (GLMM) fitted in R package glmmTMB version 1.1.1 [19]. Data exploration was carried out according to Zuur [20] to select the most appropriate error distribution and best fit models. Initial models included all variables of interest and two-way interactions. Models were evaluated by comparing Akaike’s Information Criterion (AIC) values and investigating diagnostics of scaled residuals using R package DHARMa version 0.4.1 [21]. Tests for dispersion and zero-inflation were performed using the DHARMa functions testDispersion and testZeroInflation (n=1000 simulations) and the check_overdispersion function in R package performance version 0.7.3 [22]. Significance of main effects was tested using likelihood ratio tests with the anova and drop1 functions in R (α=0.05). Non-significant interactions were dropped to retain the minimum adequate model. The final model fit was checked by visualising plots of scaled residuals against each predictor [20]. Pairwise comparisons were performed using least-squared means with Tukey’s adjustment in R package emmeans version 1.6.1 [23].

Snail abundance at 19 sites (540 observations) was analysed using a negative binomial GLMM (family = nbinom1, log link) to test the effects of snail species, habitat and sampling date. Initial models were fitted with snail count as the response variable and snail species, habitat and sampling date, and all two-way interactions, as fixed effects. Site was included as a random effect to model variation in snail abundance among sites and dependency among multiple observations over time at the same sites [20]. The final model included all main effects and an interaction between snail species and sampling date. Additionally, the overall proportions of snails found in different habitats were compared between species using a chi-square 2 × 4 contingency table of total abundance (α=0.05).

Parasitism rates of conical snails at 13 sites (360 observations) where the fly was present were analysed using a binomial GLMM with a logit link function. Initial models were fitted with parasitism rate as the response variable and snail species, habitat, sampling date, and all two-way interactions, as fixed effects. Site was included as a random effect (see above). Snail abundance (log) was included as a continuous covariate to investigate the relationship between host population size and parasitism rate. The final model included all fixed effects and the covariate with no interactions. Tests for zero inflation were non-significant; therefore, observation-level random effects were included to correct for over-dispersion in the initial model (dispersion ratio = 7.75 calculated using the check_overdispersion function) [24].

## 3. Results

### 3.1. Abundance of Conical Snails

In total, 85,673 *C. acuta* and 2412 *C. barbara* ≥5 mm were collected in 2019 and 2020 and assessed for parasitism (Table 1 and Table 2). *C. acuta* was found at all 19 sites and *C. barbara* was found at 11. Mean snail abundance differed significantly among species ($\chi^{2}=524.36$, df = 1, p<0.0001), sampling date ($\chi^{2}=83.21$, df = 3, p<0.0001) and habitat ($\chi^{2}=72.17$, df = 3, p<0.0001). There was a significant interaction between species and date ($\chi^{2}=16.31$, df = 3, p<0.001), indicating that the relationship between snail abundance and date differed between the species.

**Figure 2:**
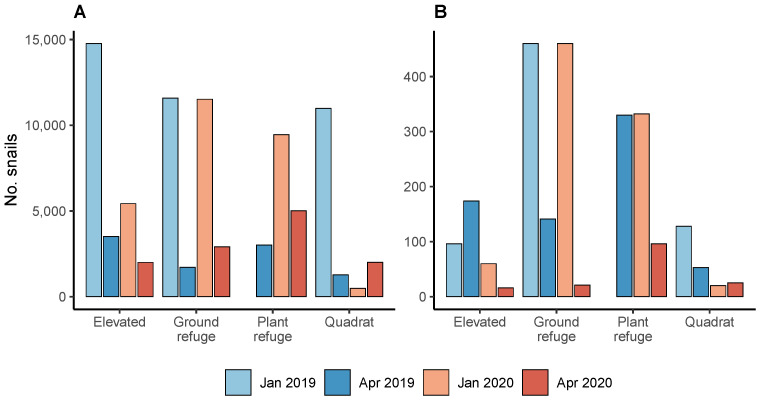
Total numbers of snails collected by habitat and sampling date for: (**A**) *C. acuta* at 19 sites ; and (**B**) *C. barbara* at 11 sites. Plant refuges were not sampled in January 2019.

The mean abundance of *C. acuta* per site and habitat declined significantly between January (mid-summer; 889.2±207.9 SEM) and April (early autumn; 126.8±13.7) in 2019 (t=−10.17, df = 1, p<0.0001) and from January (401.3±64.3) to April (165.6±24.7) in 2020 (t=−2.15, df = 1, p=0.0048) (Figure 2). In 2019, total *C. acuta* abundance declined by 76–88% in elevated, quadrat and ground refuge habitats (plant refuges were not sampled in January). In 2020, *C. acuta* abundance declined by 47–75% in elevated, plant and ground refuge habitats from January to April but increased by 314% in quadrats (Figure 2).

The mean abundance of *C. barbara* per site and habitat did not differ between January (45.6±19.5) and April (25.9±7.5) in 2019 (t=−0.75, df = 1, p=0.878), but declined significantly from January (36.3±13.7) to April (14.4±5.2) in 2020 (t=−2.67, df = 1, p=0.037). In 2020, total *C. barbara* abundance declined by 71–95% in elevated, ground and plant refuge habitats from January to April but increased by 25% in quadrats (Figure 2).

Conical snails were more abundant in elevated habitats (292.7±75.6) than in plant refuges (243.1±7.1; t=−3.29, df = 1, p=0.006) but abundance did not differ between elevated habitats and ground refuges (316.6±57.3; t=−0.37, df = 1, p=0.983). Snails were more abundant in ground refuges than plant refuges (t=−2.9, df = 1, p=0.019) and less abundant in quadrats than all other habitats (p<0.0001) (Figure 2). The proportions of snails found in each habitat differed between species ($\chi^{2}=529.35$, df = 3, p<0.0001). In total, 30% of *C. acuta* were found on elevated substrates, 32.5% in ground refuges, 20.5% in plant refuges and 17% in quadrats. By comparison, 14% of *C. barbara* were found on elevated substrates, 45% in ground refuges, 31% in plant refuges and 9% in quadrats (Figure 2).

### 3.2. Parasitism of Conical Snails by *S. villeneuveana*

**Figure 3:**
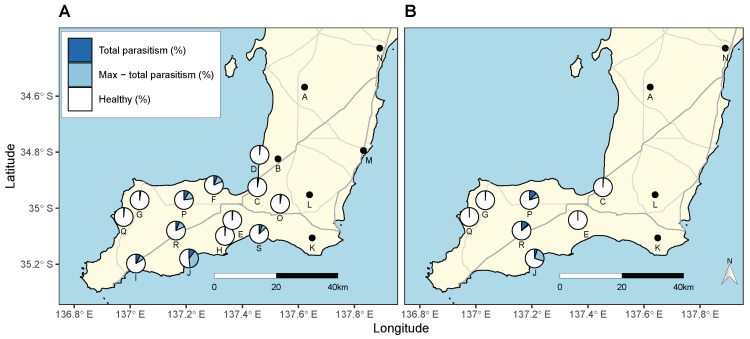
Geographic map of the Yorke Peninsula, South Australia, showing *S. villeneuveana* parasitism rates on: (**A**) *C. acuta* at 19 sites; and (**B**) *C. barbara* at 11 sites. Pies display the total parasitism rate across all sampling dates (dark blue) and the maximum parasitism rate on any single sampling date (light blue and dark blue) at sites where the fly was present. Black dots represent sites where snails were collected but *S. villeneuveana* was not recovered.

*Sarcophaga villeneuveana* was detected at 13 of 19 sites and was restricted to the southern “foot” of the Yorke Peninsula (Figure 1 and Figure 3). The fly parasitised *C. acuta* at 13 of 19 sites and *C. barbara* at 7 of 11, at distances up to 34 km from nursery release sites (site Q/release site 4) and 21 km from minor release sites (site Q/release site 14) (Figure 3).

Parasitism rates across both years were 2.27% for *C. acuta* and 3.03% for *C. barbara* at 19 sites, and 2.85% and 3.46%, respectively, at 13 sites where the fly was found (Table 1 and Table 2). The highest total parasitism rate at an individual site across all sampling occasions was 10.7% for *C. acuta* (site J) and 12.4% for *C. barbara* (site R) (Figure 3). The maximum parasitism rate at any single site and sampling occasion was 48.3% for *C. acuta* and 29.6% for *C. barbara* at site J in April 2019 (Figure 3).

Parasitism rates differed significantly among snail species ($\chi^{2}=13.78$, df = 1, p=0.0002), sampling date ($\chi^{2}=19.89$, df = 3, p=0.0001) and habitat ($\chi^{2}=60.21$, df = 3, p<0.0001). Snail abundance was a significant co-variate (estimate =−0.59±0.11, p<0.0001), implying a negative relationship between snail abundance and parasitism rate.

Parasitism rates of conical snails did not differ between years in the month of January (t=0.89, df = 1, p=0.809), but were significantly different in April (t=21.92, df = 1, p<0.0001). In 2019, parasitism increased from January (1.3%) to April (8.5%) (t=12.15, df = 1, p<0.0001) but in 2020 there was a decline from January (1.9%) to April (1.1%) (t=−8.27, df = 1, p<0.0001).

**Figure 4:**
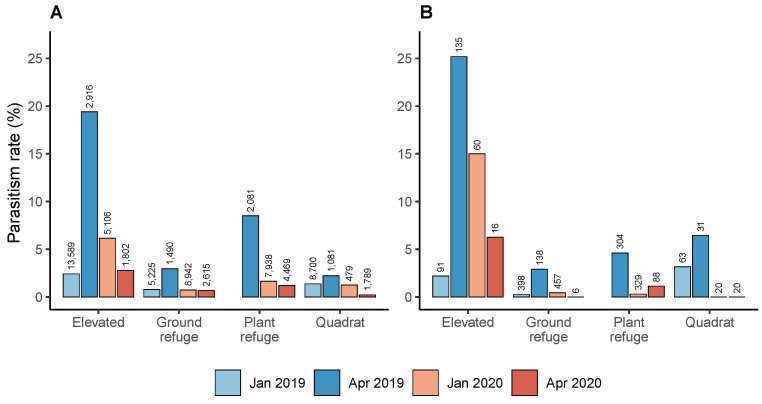
Total *S. villeneuveana* parasitism rates on: (**A**) *C. acuta* at 13 sites; and (**B**) *C. barbara* at 7 sites, by habitat and sampling date, with snail sample size shown above bars. Plant refuges were not sampled in January 2019.

Parasitism rates were higher in conical snails on elevated substrates (5.5%) than in ground refuges (0.9%) (t=6.93, df = 1, p<0.0001), plant refuges (2.5%) (t=4.32, df = 1, p<0.0001), or quadrats (1.3%) (t=6.33, df = 1, p<0.0001) (Figure 4). No difference in parasitism was found among the other habitats. In elevated substrates, the total parasitism rate was 5.4% (n=23,413) for *C. acuta* and 15.2% (n=302) for *C. barbara* (Figure 4).

## 4. Discussion

Successful biological control remains a priority for integrated management of invasive snails in Australia. To our knowledge, *Sarcophaga villeneuveana* is the only dipteran parasitoid implemented in a classical biocontrol program for terrestrial snail pests worldwide. We assessed the establishment and spread of *S. villeneuveana* 20 years after its introduction to Australia and investigated parasitism rates of conical snails in different aestivation microhabitats.

*Sarcophaga villeneuveana* has established on the southern Yorke Peninsula and dispersed at least 34 km from nursery sites where multiple fly releases were conducted from 2000–2004 [13]. The fly was not found in northern YP where low numbers of flies (n≈200 per site) were released on a single occasion. Initial surveys found a lower abundance of host conical snail populations in northern than in southern areas of the peninsula. Low populations and patchy distributions of host snails may have contributed to unsuccessful northern establishment of the fly.

The number of insects released in biological control programs can be critical for agent establishment [25,26]. The release of 1000 individuals has often been used as a guideline as the minimum release size for parasitoids, but establishment may be achieved with continuous releases of smaller numbers at several sites [27]. Leyson et al. [13] cited some difficulty rearing the parasitoid in large numbers in the laboratory, and subsequent surveys conducted 2–14 months after release did not recover the fly near Minlaton where only 200 individuals were released. In contrast, the fly was recovered within seven months after multiple releases of 300–400 per release at nursery sites near Warooka. The relatively close proximity of the four nursery sites (2–7 km) may also have contributed to successful establishment.

Overall *S. villeneuveana* parasitism of *C. acuta* was low (2.9%) across sites where the fly exists. These rates are similar to those found on the YP in 2005, 2007–08 and 2016–17 (SARDI, unpub.) and slightly less than reported in southern France (4%) from where the flies were originally sourced [7,13]. In contrast, parasitism rates of 13–25% were recorded for *S. villeneuveana* attacking *C. acuta* in southern Spain and southern Portugal [14]. Evidence for a negative relationship between snail abundance and parasitism rates requires further investigation. This result could be influenced by a small number of sites with high snail but low fly abundance in our dataset. For example, site E had the largest snail population (*n* = 23,741 conical snails collected) but a parasitism rate of only 0.1%. It seems unlikely that increased host abundance alone could inhibit fly performance, but other environmental factors may limit their ability to exploit high prey numbers in some locations.

*Cochicella barbara* was found to be a suitable host for *S. villeneuveana*, as previously reported in Europe [9]. A larger proportion of *C. barbara* were found aestivating in cryptic habitats than *C. acuta*, implying some differences in host behaviour and susceptibility to fly attack between the snail species (albeit samples sizes of *C. barbara* were much smaller). Parasitism rates were slightly higher in *C. barbara* than *C. acuta* despite smaller population sizes and a more restricted distribution on the YP. *Sarcophaga villeneuveana* attacked both snail hosts at multiple sites where *C. acuta* was the predominant species. This is an interesting finding given the smaller size of *C. barbara*. Previous studies found that larger *C. acuta* were more likely to be parasitised by *S. villeneuveana* [7,9], suggesting that the fly has a preference for larger snails or they are more suitable for development. Likewise, more flies emerged from snails in southern Iberia during spring when a greater proportion of large snails (>10 mm) were collected [14]. However, a greater proportion of smaller snails (6–10 mm) were parasitised in summer and autumn, implying that *S. villeneuveana* host usage may depend on host availability [14].

*Cochlicella acuta* in Australia belong to a genetic lineage derived from southern Iberia (Spain and Portugal) and Morocco [28]. Two common *S. villeneuveana* CO1 haplotypes from southern Portugal, Spain and Morocco are not known to occur in Australian flies, which were sourced from the Montpellier region in France [29]. Due to different geographic sources of the fly and snail hosts, it was argued that a genetic mismatch may have contributed to low parasitism success of *S. villeneuveana* in Australia [28,29].

Our study showed that *S. villeneuveana* sourced from France can perform well against conical snails in localised areas of southern Australia. Parasitism rates up to 48% for *C. acuta* and 29% for *C. barbara* were observed at sites adjacent to spring- and summer-flowering native vegetation. Nil or low parasitism was observed at locations lacking such vegetation (for example, at site E), which is common in southern Australian broad-acre farming. The success of *S. villeneuveana* in attacking conical snails may depend on the availability of floral resources. In parasitoids, consuming nectar and pollen enhances adult fitness and prolongs lifespan [30,31,32,33]. Carbohydrate-based diets with lipids and protein (e.g., pollen and nectar) maximised the lifespan and egg load of the flesh fly, *Sarcophaga crassipalpis* [34]. Thus, selecting release sites with native vegetation flowering when flies are active may enhance establishment and parasitism. Local resources may also prevent the fly from dispersing too rapidly in search of food, thereby increasing the likelihood of mating and the time spent searching for hosts [35,36]. Even a modest boost in parasitism rates soon after fly emergence in early spring may compound across successive fly generations and increase overall parasitism rates by autumn.

Parasitism increased from mid-summer to early autumn in 2019, which was expected as successive fly generations attack aestivating *C. acuta*. This snail has a mostly biennial life cycle in southern Australia and does not breed during summer [6]. However, no seasonal increase in parasitism was observed in 2020. Sampling during 21–23 April 2020 followed a rain event of ≈7 mm on 19 April 2020. Rainfall increased snail activity on the soil surface, as shown by an increase in quadrat counts as *C. acuta* and *C. barbara* exited aestivation habitats (Figure 2). It is possible that more snails were sampled as they emerged from cryptic habitats, where they are less susceptible to attack, reducing estimated parasitism.

The presence of host snails alone does not ensure establishment of *S. villeneuveana* following releases, or parasitism rates sufficient to suppress conical snail pests. Snails on elevated substrates were parasitised at higher rates than other habitats, as previously reported [7]. However, 53% of *C. acuta* and 76% of *C. barbara* aestivated in ground or plant refuges (Figure 2), suggesting these snails prefer cryptic habitats. Conical snails were observed inhabiting elevated substrates (e.g., fence posts and stubble) only when ground level refuges (plants, grasses, rocks) were unavailable. The fly is less successful attacking snails sheltering on the ground or in refuges, so reducing cryptic refuges (e.g., by controlling weeds, removing rocks and other objects) may increase parasitism as well as expose them to potentially lethal temperatures. A large decline in the abundance of live snails between January and April could reflect mortality from extreme summer heat or death of mature snails at the end of their life cycle [6]. Likewise, releasing *S. villeneuveana* near remnant patches of flowering native vegetation could provide it with food resources and shelter from heat and wind extremes.

## 5. Conclusions

The impact of the biological control agent, *S. villeneuveana*, on populations of invasive conical snails in South Australia after 20 years is limited. Factors inhibiting success may include single-fly releases with relatively few individuals, variation in abundance and distribution of host snails, cryptic snail aestivation habitats that are inaccessible to flies, a lack of floral resources for flies in agricultural areas, the use of broad-spectrum insecticides on farms that are harmful to flies, possible genetic or ecological differences between host and parasitoid populations [28,29], or a loss of genetic variability due to laboratory inbreeding [37,38]. However, evidence of substantially higher parasitism in localised environments with favourable habitat for the fly indicates the potential to improve biological control of both conical snail species. Habitat manipulation, such as the removal of snail refuges and the promotion of vegetation strips to provide floral resources for flies during spring and summer, may enhance biological control. Furthermore, *S. villeneuveana* attacks *C. barbara* at similar rates to *C. acuta*, making it suitable for release in regions where either species is a pest.

## Figures and Tables

**Table 1:** Parasitism by *S. villeneuveana* of *C. acuta* on Yorke Peninsula, South Australia, in January and April of 2019 and 2020.

	2019		2020		Total
	Jan	Apr	Jan	Apr	
No. sites with *C. acuta*	14	19	19	18	19
No. sites with fly detected	8	13	13	13	13
No. suitable snails >5 mm	37,347	9512	26,888	11,926	85,673
No. live snails	36,261	8352	23,872	8252	76,737
No. dead snails (unknown)	597	349	2499	3548	6993
No. shells with fly larva	1	15	8	1	25
No. shells with fly pupa	54	55	31	25	165
No. shells with dead fly	23	56	35	0	114
No. shells with open fly pupa	411	685	443	100	1639
No. flies emerged during rearing	104	189	69	23	385
Parasitism rate (all sites)	1.31%	8.53%	1.92%	1.06%	2.27%
Parasitism rate (fly-positive sites)	1.78%	10.72%	2.30%	1.18%	2.85%

**Table 2:** Parasitism by *S. villeneuveana* of *C. barbara* on Yorke Peninsula, South Australia, in January and April of 2019 and 2020.

	2019		2020		Total
	Jan	Apr	Jan	Apr	
No. sites with *C. barbara*	5	9	11	5	11
No. sites with fly detected	3	5	7	4	7
No. suitable snails >5 mm	684	698	872	158	2412
No. live snails	638	636	820	124	2218
No. dead snails (unknown)	41	8	40	32	121
No. shells with fly larva	0	1	1	0	2
No. shells with fly pupa	0	5	1	1	7
No. shells with dead fly	0	2	2	0	4
No. shells with open fly pupa	5	46	8	1	60
No. flies emerged during rearing	1	9	0	0	10
Parasitism rate (all sites)	0.73%	7.74%	1.38%	1.27%	3.03%
Parasitism rate (fly-positive sites)	0.91%	8.88%	1.39%	1.54%	3.39%

## Data Availability

The data presented in this study are available on request from the corresponding authors.
